# Direct RNA Sequencing for the Study of Synthesis, Processing, and Degradation of Modified Transcripts

**DOI:** 10.3389/fgene.2020.00394

**Published:** 2020-04-28

**Authors:** Mattia Furlan, Iris Tanaka, Tommaso Leonardi, Stefano de Pretis, Mattia Pelizzola

**Affiliations:** ^1^Center for Genomic Science, Istituto Italiano di Tecnologia, Milan, Italy; ^2^Department of Physics, National Institute of Nuclear Physics, University of Turin, Turin, Italy

**Keywords:** RNA modification, m^6^A, direct RNA sequencing, metabolic labeling, nascent RNA, RNA metabolism, long reads sequencing, nanopore

## Abstract

It has been known for a few decades that transcripts can be marked by dozens of different modifications. Yet, we are just at the beginning of charting these marks and understanding their functional impact. High-quality methods were developed for the profiling of some of these marks, and approaches to finely study their impact on specific phases of the RNA life-cycle are available, including RNA metabolic labeling. Thanks to these improvements, the most abundant marks, including N^6^-methyladenosine, are emerging as important determinants of the fate of marked RNAs. However, we still lack approaches to directly study how the set of marks for a given RNA molecule shape its fate. In this perspective, we first review current leading approaches in the field. Then, we propose an experimental and computational setup, based on direct RNA sequencing and mathematical modeling, to decipher the functional consequences of RNA modifications on the fate of individual RNA molecules and isoforms.

## Introduction

More than a 100 RNA modifications have been identified since the 1950s (Boccaletto et al., 2018). They were first observed in abundant populations of non-coding transcripts (e.g., tRNAs) and in a second moment, due to the improvement of profiling techniques, their pervasive presence was confirmed in coding transcripts (Roundtree et al., 2017). Different modifications were found to co-occur on the same RNA molecule (Jackman and Alfonzo, 2013). In some cases, rather than a mere stochastic effect due to the modification frequency, their co-occurrence suggested reciprocal regulation mechanisms (Xiang et al., 2018).

The N^6^-methyladenosine (m^6^A) emerged as one of the most abundant modifications of coding transcripts (Roundtree et al., 2017), and it was shown to be involved in the regulation of various biological processes, including cellular differentiation (Lin and Gregory, 2014; Wang Y. et al., 2014; Chen et al., 2015; Geula et al., 2015; Zhang et al., 2017a), meiosis (Bushkin et al., 2019), heat stress response (Zhou et al., 2015), gametogenesis (Wojtas et al., 2017), and neurons activity (Engel et al., 2018). Furthermore, aberrant m^6^A patterning was shown to be associated with diseases insurgence and progression (Tong et al., 2018; Ianniello et al., 2019; Yang et al., 2019). A number of effectors were identified that are responsible for m^6^A deposition (e.g., METTL3 and METTL14) (Liu et al., 2014; Ping et al., 2014; Schwartz et al., 2014), recognition (e.g., members of the YTH domain family) (Luo and Tong, 2014; Xu et al., 2014; Zhu et al., 2014; Xiao et al., 2016), and removal (FTO and ALKBH5) (Jia et al., 2011; Zheng et al., 2013), suggesting that this mark could be dynamically regulated. Genome-wide m^6^A profiling, through immunoprecipitation with m^6^A-specific antibodies followed by short-reads RNA sequencing (srRNA-seq), revealed the preferential, while not exclusive, association of the mark with the central adenosine in the RRACH sequence context around the stop codon of messenger RNAs (R = G or A and H = A, C, or U) (Dominissini et al., 2012; Meyer et al., 2012). Notably, m^6^A marks have been linked to different biological processes depending on their relative position within a transcript, suggesting a context-specific role for this mark (Shi et al., 2019). However, we have only started revealing the rules that determine the preference of the mark for specific bases, and their impact on specific downstream biological processes (Yue et al., 2018). Altogether, m^6^A was identified as a key determinant of RNA decay (Wang X. et al., 2014) and translation (Wang et al., 2015), while discordant reports were published about its involvement in splicing regulation (Haussmann et al., 2016; Xiao et al., 2016; Bartosovic et al., 2017; Ke et al., 2017; Darnell et al., 2018; Kasowitz et al., 2018; Louloupi et al., 2018).

RNA metabolic labeling (Dolken et al., 2008) emerged as a powerful approach that not only allows to characterize the association of m^6^A, or other RNA modifications, with nascent transcripts, but also allows to quantify the impact of these marks on the dynamics of all key steps of the RNA life cycle, and specifically on the kinetic rates of RNA synthesis, processing, and degradation. The application of this technique confirmed the role of m^6^A on the regulation of RNA stability, and suggested its influence on the dynamics of RNA synthesis and processing (Furlan et al., 2019b).

The application of the current leading approaches for profiling RNA modifications, such as m^6^A, generated important findings about the functional role of these marks (Roundtree et al., 2017). However, these approaches are heavily based on srRNA-seq, and are afflicted by a number of downsides: different methods were developed for various modifications, they only allow to indirectly map the targeted mark, they are poorly suitable for analyses at the level of single molecules and isoforms, they cannot be readily used to profile co-occurring modifications, and they are difficult to be paired with RNA metabolic labeling. In this perspective, we discuss how direct RNA sequencing (such as nanopore-based sequencing of native RNAs) is rapidly emerging as a powerful alternative approach, which has the potential to overcome these issues, bursting the field of epitranscriptomics.

## Experimental and Computational Approaches for the Quantification of RNA Kinetic Rates

**FIGURE 1:**
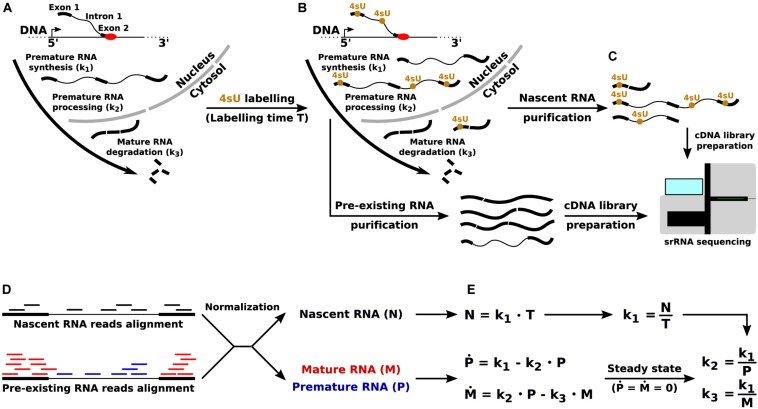
Quantification of the RNA kinetic rates through RNA metabolic labeling coupled with srRNA-seq. **(A)** The key steps of the RNA life cycle, and the corresponding RNA kinetic rates: synthesis (k_1_) of premature RNA, processing (k_2_) of premature into mature RNA, and degradation (k_3_) of mature transcripts. **(B)** Incorporation of the uridine analog 4sU into newly synthetized transcripts. **(C)** Pre-existing and nascent RNA purification and sequencing through srRNA-seq. **(D)** Quantification of premature (P), mature (M), and nascent (N) RNA from srRNA-seq reads. **(E)** RNA life cycle mathematical modeling and quantification of the RNA kinetic rates in the steady-state limit.

The state of the art approach to infer the kinetic rates governing the RNA life cycle – synthesis of premature RNA, its processing into mature RNA, and the degradation of the latter – is based on the joint quantitative analysis of total and nascent RNA (Figure 1). While the former is simply obtained through RNA-seq, the latter can be profiled through RNA metabolic labeling. In this technique, a nucleotide carrying an exogenous modification (e.g., 4-thiouridine, 4sU) is provided in the cells’ medium, and is incorporated into nascent transcripts during the labeling time. Thus, the presence of the exogenous modification can be used for the physical (Dolken et al., 2008) or *in silico* (Baptista and Dölken, 2018) separation of newly synthetized transcripts from pre-existing ones.

Mathematical modeling is then used for the gene-level quantification of RNA kinetic rates, for example as implemented and documented in the INSPEcT R/Bioconductor library (de Pretis et al., 2015; Furlan et al., 2019a). Briefly, when short labeling times are adopted (<1 h), the quantification of nascent RNA for each gene provides a proxy for the rate of synthesis of premature RNA. Then, total RNA-seq reads are used to measure the abundance of premature and mature transcripts: reads that entirely map to one or more exons are used to quantify mature RNA species, and the remaining mapped reads (entirely, or partially, covering introns) are used for the quantification of premature species. Finally, the combination of synthesis rate and premature RNA abundance is used to quantify the rate of processing, while the combination of synthesis rate and mature RNA abundance allows the quantification of degradation rates (Furlan et al., 2019a).

The joint analysis of the information gained from RNA metabolic labeling experiments, together with the profiling of specific RNA modifications, would be extremely powerful for the study of the functional consequences of these marks on specific RNA life cycle steps. However, while the application of metabolic labeling for the profiling of nascent RNA (Dolken et al., 2008) and for the quantification of the RNA kinetic rates (Dolken et al., 2008; Miller et al., 2011; Rabani et al., 2011, 2014; de Pretis et al., 2015; Furlan et al., 2019a) is an established approach, its combination with the profiling of RNA modifications is more problematic. In fact, the joint profiling of nascent and modified RNA requires the identification of at least two RNA modifications: the endogenous mark (e.g., m^6^A), and the exogenous modification used for the labeling (e.g., 4sU). As we discuss in the following sections, this is a complex task that can be only indirectly implemented through current approaches.

## Detection of Rna Modifications Through Short-Reads RNA Sequencing

Numerous protocols based on srRNA-seq were developed for the identification of either endogenous (e.g., m^6^A) or exogenous (e.g., 4sU) RNA modifications. A first class of methods is based on the enrichment of modified RNAs before the sequencing. This relies either on the use of specific antibodies [e.g., MeRIP-seq for m^6^A detection (Dominissini et al., 2012; Meyer et al., 2012)], or the use of enzymes involved in the metabolism of the modification [e.g., tRNA methyltransferase DnmA (Muller et al., 2013)], or on the availability of tags such as biotin on the modified residues [e.g., 4sU-based RNA metabolic labeling (Dolken et al., 2008)]. These techniques do not provide neither the exact modification site (they are limited to 100–200 bp resolution), nor a precise quantification of the proportion of modified transcripts (Molinie et al., 2016), despite the development of *ad hoc* experimental (Sun et al., 2012) and computational (de Pretis et al., 2015) normalization techniques. Indeed, an alternative approach, m^6^A-LAIC-seq (Molinie et al., 2016) has been developed that relies on spike-ins to provide a precise quantification of the m^6^A abundance, at the cost of skipping the RNA fragmentation step and losing positional information on the mark. A second class of methodologies is based on the identification of RNA modifications signatures in the retro-transcribed cDNA. One approach belonging to this class exploits the early interruption of retrotranscription at the modification site to produce specific truncation signatures [e.g., ICE-seq for inosine detection (Sakurai et al., 2010)]. Alternative approaches were developed to retro-transcribe the modified bases and their native counterparts to different nucleotides, thus inferring the site of the modification based on specific mismatches in the reads alignment (Baptista and Dölken, 2018). For example, SLAM-seq allows the *in silico* identification of reads derived from nascent RNAs by inducing the pairing of alkylated 4sU to guanines (Herzog et al., 2017). These methods markedly increase the resolution, but are typically semi-quantitative, suffering from low sensitivity (Neumann et al., 2019). Hybrid techniques were also developed. For example, methylation induced cross-linking and immunoprecipitation (miCLIP) combines m^6^A-immunoprecipitation with the antibody cross-linking, leading to conversion and truncation events. Their identification in the sequencing results allows the mapping of m^6^A at base-resolution (Linder et al., 2015). However, this method is affected by low crosslink efficiency, reducing the sensitivity. Recently, two novel approaches were developed that do not rely on immunoprecipitation. MAZTER-seq (Garcia-Campos et al., 2019) allows the quantitative and base-resolution identification of m^6^A marks, relying on the use of a restriction enzyme that cuts only when the target site is not methylated. As a downside, the mapping is limited to the identification of m^6^A marks in specific context sites (16% of all expected m^6^A sites in mammals). DART-seq (Meyer, 2019) recruits APOBEC1 proteins at m^6^A sites through readers of the YTH family, allowing the identification of the marks by the detection of adjacent C to U mutations. It was used in combination with srRNA-seq, with as little as 10 ng of total RNA, and with long-reads RNA sequencing (lrRNA-seq), leading to single transcript m^6^A detection. The key downside of this method is the required cells transfection with APOBEC1-YTH fusion protein. Finally, the ability to quantify the abundance of m^6^A marks remains to be established.

A number of computational tools were developed that are useful for calling RNA modifications on srRNA-seq data, especially tailored toward the analysis of m^6^A marks in MeRIP-seq datasets. exomePeak, while not originally developed for this task, is one of the most frequently adopted tools for the identification of m^6^A peaks (Meng et al., 2013). Indeed, a detailed protocol was described for its application on MeRIP-seq datasets (Meng et al., 2014). This tool adopts a sliding window approach with a conditional test relying on Poisson distributions. HEPeak is an HMM-based tool dedicated to the identification of m^6^A marks, claiming improved sensitivity and specificity compared to exomePeak (Cui et al., 2015). From the same authors, MeTPeak was later proposed that is able to take advantage of the variance across replicates, and models the reads dependency across a region (Cui et al., 2016). A number of tools were developed that are dedicated to differential RNA methylation analysis, including MeTDiff (Cui et al., 2018), FunDMDeep (Zhang S. Y. et al., 2019), and RADAR (Zhang Z. et al., 2019). Finally, m^6^A viewer is a Java stand alone application that supports detection, analysis, and visualization of m^6^A marks, the former relying on the previously described tools (Antanaviciute et al., 2017).

Besides the specific limitations of each technique, all available protocols for the profiling of RNA modifications through srRNA-seq share some key limitations. *First*, they require specific reagents for each modification of interest, which currently limits the profiling to a handful of modifications (Helm and Motorin, 2017). *Second*, the library preparations, and the sequencing procedure, remove the RNA marks. As a consequence, most available approaches for the modifications profiling are indirect, reducing specificity and sensitivity (Helm and Motorin, 2017). *Third*, the reduced length of srRNA-seq reads (50–300 bp) is a major obstacle for the analysis of individual RNA molecules, despite the development of methods to infer isoforms expression from these data (Zhang et al., 2017b). As a consequence, the assignment of individual or co-occurring modifications to a given RNA molecule, or even to a given isoform, is not feasible. *Fourth*, srRNA-seq protocols are not readily applicable to detect two (or more) RNA modifications simultaneously.

Although recent interesting technical advances are starting to appear [e.g., simultaneous detection of N^1^-methyladenosine, 5-methylcytosine, and pseudouridine (Khoddami et al., 2019)], these methods highly depend on the specific combination of marks. The reasons for this limitation are manifolds. Likely, the methods for the profiling of different modifications should be consecutively applied, and the output of one method could be poorly suitable for the subsequent. For the same reason, a high amount of starting material is likely to be necessary, to avoid capturing only highly expressed transcripts. Alternatively, numerous rounds of PCR would be necessary, introducing amplification biases (Aird et al., 2011; Kebschull and Zador, 2015). The limitations in specificity and sensitivity of each method would combine. Moreover, it would be crucial and cumbersome to develop normalization procedures for the comparison of the results from each approach, possibly based on spike-ins. Finally, it would be hard to keep track of the positional information of each modification.

**FIGURE 2:**
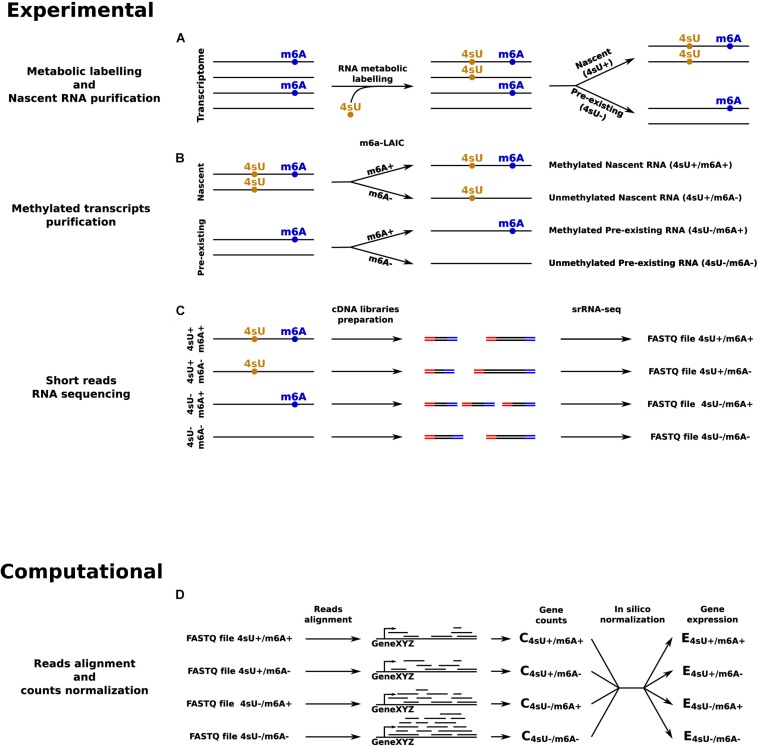
srRNA-seq based approach to quantify transcripts’ expression levels in all the four possible combinations given by the presence or absence of 4sU and m^6^A RNA modifications. **(A)** RNA metabolic labeling, based on the incorporation of 4sU, is applied to separate the nascent portion of the transcriptome from the pre-existing counterpart. **(B)** m^6^A-LAIC-seq is applied for both nascent and pre-existing RNAs to separate methylated from unmethylated transcripts. **(C)** cDNA library preparation and sequencing for: pre-existing unmethylated RNAs, pre-existing methylated RNAs, nascent unmethylated RNAs, and nascent methylated RNAs. **(D)** *In silico* reads alignment, counts quantification, and normalization to estimate transcripts’ expression levels across all the four conditions.

Things would get even more complicated when, in addition to the mark of interest, the dynamics of RNA metabolism are also of interest, which require the identification of an exogenous modification as second mark. In this case, to quantify the RNA kinetic rates of modified and unmodified RNAs, it would be necessary to quantify all four possible combinations: nascent/modified, nascent/unmodified, pre-existing/modified, and pre-existing/unmodified transcripts (Figure 2). Currently, the best approach to jointly identify 4sU and m^6^A would be to start by separating nascent and pre-existing RNA using 4sU metabolic labeling and purification (Dolken et al., 2008). Then, for each of these, the m^6^A-LAIC-seq protocol could be applied to separate m^6^A methylated RNAs from unmethylated transcripts. At the end, four samples per condition should be prepared and sequenced. This approach is evidently very complex and onerous, it would require a lot of starting material and complicated downstream analyses, including spike-ins based normalization of the datasets. For all these reasons, the most common compromise is to profile m^6^A, and to perform metabolic labeling through independent experiments (Li et al., 2017; Furlan et al., 2019b). However, this type of approach completely compromises the possibility of a direct quantification of the dynamics of modified and unmodified transcripts, since it only allows to quantify the dynamics of the pool of transcripts for each gene, and then combine this information with the expected degree of modification for that population. Altogether, approaches based on srRNA-seq are increasingly inadequate and could hamper the progress in the field of epitranscriptomics.

## Long-Reads Direct Rna Sequencing for the Identification of Modifications in Native RNAs

In the last few years remarkable efforts were dedicated to overcoming the limitations of srRNA-seq based approaches (Stark et al., 2019) for the identification of RNA modifications within individual RNA molecules and isoforms. As a result, few novel sequencing approaches that emerged recently allow rRNA-seq. One platform, PacBio (developed by Pacific Biosciences), exploits a sequencing by synthesis approach mediated by an immobilized polymerase (Eid et al., 2009). Another one, which will be the main focus in the next sections of this perspective, was developed by Oxford Nanopore Technologies (ONT), and consists of an array of thousands of nanopores which allow a flow of ions across a dielectric membrane, thus generating a measurable current. The active translocation of a molecule of nucleic acids (either DNA, cDNA, or RNA) through each pore, mediated by an engineered motor protein, results in a sequence-specific perturbation of the measured current. In turn, this signal can be exploited to infer the corresponding sequence of nucleotides (Kasianowicz et al., 1996; Smith et al., 2015). lrRNA-seq approaches were successfully used to study transcriptional and post-transcriptional regulation in various physiological and disease conditions (De Roeck et al., 2017; Aneichyk et al., 2018; Anvar et al., 2018; Nattestad et al., 2018), including single-cells (Byrne et al., 2017). Focusing on RNAs, these techniques can produce single reads of up to 10^4^ bases, with an average length of almost 1 Kb for ONT (Workman et al., 2018). Hence, in a number of cases, this allows the profiling of full-length RNA molecules, and the fine characterization of their alternative isoforms. This is especially true for mature transcripts, whose median length for human and mouse mRNAs is around 2 Kb [based on the hg19 and mm10 UCSC genome releases (Haeussler et al., 2019)]. Instead, the likelihood of sequencing full-length premature transcripts is lower. Indeed, their median open reading frame length is in the 13–18 Kb range, although co-transcriptional splicing could significantly reduce this figure (it is likely that some intron was already excised before the completion of RNA synthesis).

The direct RNA sequencing approach developed by ONT does not go through the conversion of RNA into cDNA, and does not rely on amplification steps. For these reasons, the RNA modifications are preserved and can induce specific alterations in the current registered by the sequencer (Garalde et al., 2018). Altogether, this approach represents a potential solution to most of the limitations of srRNA-seq discussed above, due to its ability to directly identify any, and possibly multiple, RNA modification in single, full-length molecules. dRNA-seq was recently applied to study the transcriptome of viruses (Moldován et al., 2018; Tombácz et al., 2018; Boldogkõi et al., 2019; Depledge et al., 2019), yeast (Garalde et al., 2018), animals (Jiang et al., 2019; Roach et al., 2019; Smith et al., 2019), and plants (Zhao et al., 2019).

However, a number of limitations characterize the young field of dRNA-seq. *First*, current dRNA-seq protocols are available only for the sequencing of targeted, non-polyadenylated RNAs (Keller et al., 2018; Smith et al., 2019) or polyadenylated RNAs. This is due to the library preparation protocolos, which typically targets polyA tails or specific 3′ sequences for ligating sequencing adapters anchoring the motor protein. This limitation could be addressed using adapters with random 3′ sequences, with the risk of introducing a bias for recurrent RNA motifs, or through *in vitro* polyadenylation of transcripts devoid of a polyA-tail (Wongsurawat et al., 2018). *Second*, while the throughput of dRNA-seq is rapidly growing, it currently compares to the low- or mid-end coverage of srRNA-seq experiments. This could limit the number of detectable transcripts, although, importantly, the abundance of those that can be detected is well correlated with high-coverage srRNA-seq data (Garalde et al., 2018). This issue could be solved in the future by improving the speed of translocation of RNAs across the nanopore, and/or extending the sequencing time by prolonging the pores’ lifetime. Noteworthy, given the same throughput in terms of sequenced bases, lrRNA-seq vs srRNA-seq data have a substantial difference: while the former allows detecting entire transcripts, the latter offers a more unbiased sampling of any RNA fragment, thus also covering a larger portion of the transcriptome (Soneson et al., 2019). This could in part be obviated by a coarse RNA fragmentation before the library preparation, and would also reduce the 3′ coverage bias of dRNA-seq data, whose reads start from a transcript’s 3′ end. A drawback of this approach is that it would compromise the one-to-one correspondence between reads and RNA molecules. *Third*, the accuracy of base calling on dRNA-seq data is currently significantly lower than srRNA-seq. When base calling errors occur at sites of RNA modification, they are likely due to the inability of the base caller’s to deal with changes in the signal originated by those marks. However, these errors represent a small fraction of incorrect base calls, due to the low number of marks per transcripts (e.g., 2–3 m^6^A marks per RNA). Hence, reduced base calling accuracy is not considered a major issue in the field of RNA modifications but, on the contrary, represents an opportunity for aiding the identification of modified bases (Liu et al., 2019). *Fourth*, there could be limitations on the detectability of specific RNA modifications. For example, in the context of RNA metabolic labeling, the ability of dRNA-seq to identify various (exogenous) modified nucleotides was tested (Maier et al., 2019). This revealed that 4sU modified nucleotides, commonly used in metabolic labeling through srRNA-seq, were not compatible with the nanopores, leading to blockages during the sequencing, although this issue was not confirmed in a more recent report (Drexler et al., 2019). Instead, other marks, such as 5-ethynyluridine (5eU), were found to be suitable for these experiments.

In conclusion, this is a young and rapidly evolving research field, based on a highly collaborative research community. Hence, numerous labs are actively involved to find solutions or improvements to all these limitations, which are likely to be fully or partially overcome in the next few years (Rang et al., 2018).

## Computational Tools for the Detection of Modifications in Long-Reads Direct RNA Sequencing

Recent and growing literature is available about the footprints left by RNA modifications on dRNA-seq data, and how to exploit them to detect RNA marks (Xu and Seki, 2019). Differences in current levels between native bases and their modified counterparts were reported for m^6^A, m^5^C, m^7^G, and pseudouridine (Garalde et al., 2018; Workman et al., 2018; Smith et al., 2019). Moreover, the increase of base miscalls frequency in concomitance to modified sites were observed next to “A-to-I,” 7-methylguanosine and pseudouridine sites (Workman et al., 2018; Smith et al., 2019). These observations led to the development of specific computational tools for the detection of RNA modifications.

Tombo, an official tool provided by ONT, requires a model of the signal generated by the modification in all possible sequence contexts, to be used as a baseline for the identification of the same mark at single molecule resolution within a new dRNA-seq dataset (Stoiber et al., 2016). Notably, baseline data for 5-methylcytosine marks are included in the tool (Viehweger et al., 2019). Alternatively, data for a condition devoid of modifications can be provided. With a similar approach, Tombo was recently used to identify m^6^A in yeast with an accuracy of 69% and a recovery of 59%, compared with m^6^A peaks identified with MeRIP-seq (Liu et al., 2019). Obviating for the need of these positive or negative baseline data, Tombo can be used to compare the signal observed for each k-mer with that of any possible unmodified k-mer, although this approach is affected by high false positive rates.

EpiNano relies on a support vector machine, and exploits the increased frequency of alignment errors and the low base quality caused by the presence of the modification of interest (Liu et al., 2019). The tool is first trained and tested on two sets of *in vitro* transcribed synthetic RNAs that contain either m^6^A only or unmodified adenosine only. Its classification performance in the context of the expected m^6^A RRACH motif was excellent (area under the curve up to 0.944). Rather, the performance decreased when the tool was applied on *in vivo* yeast data and benchmarked with MeRIP-seq m^6^A calls for the same conditions (accuracy: 87% and recovery: 32%). In terms of downsides, EpiNano requires prior knowledge on the sequence motif for the mark of interest, and it cannot achieve single molecule resolution, since it aggregates the information derived from multiple reads alignments.

ELIGOS aims at the unbiased identification of any RNA modification that would impact bases errors frequencies. It relies on the comparison between dRNA-seq of native and cDNA-converted transcripts, the latter used as a reference that is devoid of any mark due to the retro-transcription to cDNA (Wongsurawat et al., 2018). ELIGOS was tested on *in vitro* fully modified transcripts, rRNAs from various species, and a human lymphoblastoid cell line. Like Tombo, the main downside of ELIGOS is in terms of false positive rates.

A further method for m^6^A identification that was recently released is called MINES (Lorenz et al., 2019). This software implements a random forest classifier trained on a set of high confidence, experimentally defined, m^6^A sites within canonical DRACH motifs. This method showed high accuracy and precision, and also has single-isoform, single-base resolution. However, MINES can only predict m^6^A sites within DRACH motifs, which only comprise a portion of all m^6^A sites. A further potential limitation is due to the fact that the classifier was trained on m^6^A sites defined with CLIP and – as such – might suffer of biases similar to those caused by antibody-based methods.

Nano-ID was recently developed for detecting the incorporation of the exogenous mark 5eU into nascent RNA (Maier et al., 2019), implementing the analysis of RNA metabolic labeling on the ONT platform. This tool relies on a neural network trained to distinguish dRNA-seq signal of fully unlabeled from fully labeled RNAs (24 h 5eU labeling time), to classify reads from nascent transcripts, while no positional information on 5eU marks is returned. The results achieved by nano-ID on this test set were very encouraging (area under the curve 0.95), and the tool was applied to infer the isoform-level rates of synthesis and degradation in K562 cells, and how they were affected by heat shock.

Nanocompore is a novel tool recently released, which is based on the comparison of a condition of interest with a condition where the writer for a specific mark was depleted or removed (Leger et al., 2019). The idea is that the removal of the mark leads to a change in the ONT signal, which could be identified through statistical tests by comparing the two conditions. As a result, Nanocompore can provide near base-resolution and single molecule calls for the mark of interest. Alternatively, analogously to ELIGOS, if the baseline condition is depleted of multiple or possibly all marks (e.g., via *in vitro* transcription), the tool returns the corresponding changes in the signal to identify all marks occurrence, while mark-specific calls are not possible. Advantages and disadvantages of the tools discussed above are reported in Table 1.

**TABLE 1:** Comparing strengths and pitfalls of four software packages for m^6^A detection from Nanopore dRNA-seq data.

	EpiNano	ELIGOS	MINES	Nanocompore
Requires training dataset	Yes	No	Yes	No
Requires comparison condition	No	Yes (cDNA)	No	Yes
Limited to RACH motifs	Yes	No	Yes	No
Single nucleotide resolution	Yes	Yes	Yes	No
Isoform resolution	Yes	Yes	Yes	Yes
Single molecule resolution	No	No	No	Yes
Able to distinguish different modifications	Yes	No	Yes	Yes

## Applying Direct Rna Sequencing to Quantify the Dynamics of Modified RNAs

**FIGURE 3:**
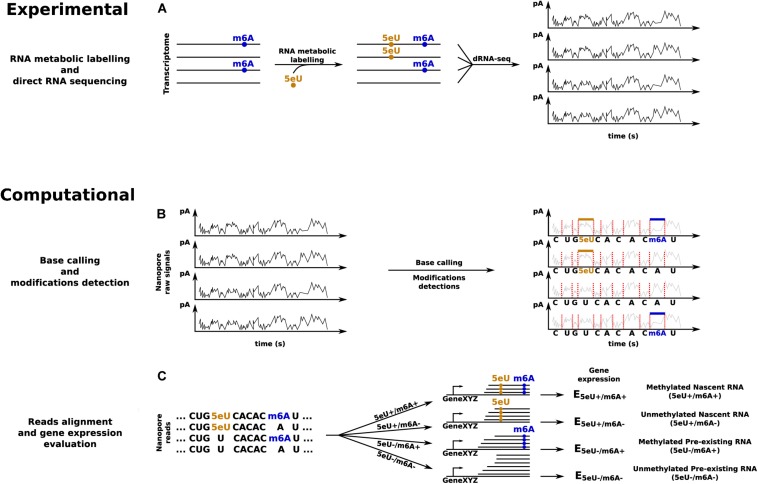
dRNA-seq based approach to quantify transcripts’ expression levels in all the four possible combinations given by the presence or absence of 5eU and m^6^A RNA modifications. **(A)** RNA metabolic labeling, based on the incorporation of 5eU, is applied to mark nascent transcripts, before direct RNA sequencing. **(B)** Base calling and identification of the two RNA modifications. **(C)** Reads alignment and *in silico* separation, according to the presence or absence of each RNA modification, to estimate transcripts’ expression levels across all the four conditions.

The recent surge in the number of tools for the identification of specific modifications indicates that the field is quickly progressing. However, a number of improvements are required for the joint analysis of the patterning of an endogenous modification, such as m^6^A, with the quantification of the corresponding RNA dynamics, via metabolic labeling and profiling of exogenous modifications such as 4sU or 5eU (Figure 3).

*First*, the modifications have to be profiled at single molecule resolution, a prerequisite for the direct matching of the RNA dynamics with the modification status. This would allow understanding how the RNA kinetic rates are impacted by the presence of a modification, and, potentially, by its patterning (numerosity and position). Notably, the frequency and the specific position of occurrence of the marks is increasingly recognized as an important factor. For example, the fate of RNAs carrying multiple m^6^A marks was shown to be influenced by a liquid–liquid phase separation processes driven by the binding of readers of the YTH family. Eventually, those transcripts were shown to be targeted to specific cellular compartments, including stress-granules and P-bodies, with important consequences for their translation and stability (Ries et al., 2019).

*Second*, tools based on supervised machine learning could be preferable in the field, compared to methods for the unsupervised identification of the marks. In fact, various confounding factors could potentially affect direct RNA sequencing data, which could be easier to address in a supervised framework. However, supervised methods require training on sets of modified transcripts, which should be built so that they closely reflect the characteristics of *in vivo* datasets. For example, for endogenous modifications, rather than producing *in vitro* fully modified transcripts, the level of modification could be tuned by mixing unmodified and modified nucleotides to match the expected frequency of the mark. For exogenous marks, the approach described in Maier et al. (2019) could be followed, where physiological high-level of incorporation of a modified nucleotides are obtained by its prolonged availability in the cells medium.

*Third*, the current ONT signal (amplitude and dwell time) is the most direct data type for the identification of the marks, compared to more indirect measurements, such as the error rate. While tools, such as EpiNano, showed a good performance by only using the latter, we would recommend trying to incorporate information from the former. Indeed, indirect measurements could be completely or partially originated by unexpected causes, which could lead to high false positive rates with *in vivo* datasets.

*Fourth*, the quantification of RNA dynamics should include the step of premature RNA processing. This is often neglected, by assuming the corresponding rate being constant. However, RNA synthesis and processing are tightly coupled, then when the former is modulated, which often occurs, the latter is also expected to be altered (Neugebauer, 2019). Moreover, recent reports start unveiling the frequency and importance of changes in splicing dynamics (Rabani et al., 2014; de Pretis et al., 2015, 2017; Louloupi et al., 2018; Furlan et al., 2019a; Wachutka et al., 2019). The cost of considering the processing step is two fold: it markedly increases the complexity of the underlying mathematical models, and implies the quantification of the abundance of premature RNA species. The latter is specifically problematic for the ONT platform. Indeed, the library preparation procedure expects transcripts with the polyA tail, which are lacking in premature RNAs. *In vitro* polyadenylation with m^6^A could be used for adding m^6^A-tails to premature transcripts. This would allow the sequencing of premature RNAs, and would preserve the sequencing information about the endogenous tails of mature transcripts, for studies on their functional impact on RNA dynamics.

*Fifth*, reads from premature RNAs would have to be distinguished from those from mature species. The presence of an endogenous polyA tail would provide a way to computationally identifying reads from mature species. However, this approach would fail for those mRNAs that are not polyadenylated in their endogenous mature form. An alternative criterion is to consider the reads containing introns as premature RNA. This could be problematic in case of intron retention, which in many organisms, including humans, is not infrequent (Chaudhary et al., 2019; Monteuuis et al., 2019). The request of more than one intron in order to classify a read as premature RNA would probably eliminate this issue. Of course, such a strict condition would cause the exclusion of those genes that have less than two introns, which often occurs in some organisms (e.g., yeast or plants). The best criterion could eventually be a mix of the proposed approaches, selected according to the biological system under analysis and the transcripts of interest. For instance, to study mRNA kinetics in mammalian cells, mature RNA could be estimated considering fully spliced, polyadenylated transcripts, while premature RNA could be quantified from the remaining reads, possibly requiring the presence of one or more introns.

Once proficient algorithms for the detection of the endogenous (e.g., m^6^A) and exogenous (e.g., 5eU) marks at single molecule resolution are in place, they could be used, in series, for the identification of the four possible classes defined by the presence or absence of each modification. The performance of such an approach should be tested on a dataset generated *ad hoc*. The genesis of reads with both the RNA modifications, or missing only the exogenous mark, is feasible by using or avoiding long-time metabolic labeling, respectively. Instead, reads devoid of both the base analogs can be produced sequencing the corresponding cDNA. It is more difficult to generate transcripts that lack only the endogenous modification, which could be obtained by knocking-out the corresponding writer (for those marks for which this is known). However, genetic compensation (El-Brolosy and Stainier, 2017) or writer’s redundancy could lead to the incomplete depletion of the RNA modification.

## Additional Remarks

The study of the impact of RNA modifications on the RNA life cycle dynamics would largely benefit from the development of a unified computational framework. This, starting from long reads dRNA-seq data, should manage the RNA kinetic rates inference, according to their modification status, at the level of individual transcriptional units or specific isoforms.

A convenient starting point could be INSPEcT (de Pretis et al., 2015), a tool developed in our lab for the inference of all RNA kinetic rates (synthesis, processing, and degradation) from srRNA-seq data. The user should only pay attention to quantify premature and mature RNA in both nascent and pre-existing fractions according to the guidelines presented above. Additionally, if the quantification of dynamics at the level of specific isoforms is desired, the analysis should be conducted considering the reads associated with each isoform, rather than those associated with the whole transcriptional unit. Finally, if this analysis is applied independently on the set of modified and unmodified reads, it would allow comparing the kinetic rates among them, as illustrated in Figure 3B.

INSPEcT has been recently extended by implementing a novel approach that allows the inference of synthesis, processing and degradation kinetic rates without nascent RNA profiling (Furlan et al., 2019a). This approach could be an interesting alternative to study the relation between RNA modifications and RNA life cycle dynamics without requiring metabolic labeling and the consequent identification of the exogenous modification. This would also allow studying the impact on RNA dynamics of those modifications that mark the same base targeted by metabolic labeling, such as pseudouridine and 5eU.

In conclusion, a number of recent and on-going technology advancements are significantly facilitating the study of the functional consequences of RNA modifications on the fate of marked transcripts. In particular, the combined application of RNA metabolic labeling, for the profiling of nascent transcripts and the quantification of the kinetic rates governing the RNA life cycle dynamics, and of long-reads direct RNA sequencing, is particularly promising. Indeed, they promise to deliver data of unprecedented quality and resolution, and should allow studying the impact of RNA modifications at the level of individual molecules and isoforms.

## Author Contributions

MF and MP conceived the study. MF led the writing and produced the figures. MP supervised the study and the writing of the manuscript. All authors contributed discussing and writing the manuscript.

## Conflict of Interest

The authors declare that the research was conducted in the absence of any commercial or financial relationships that could be construed as a potential conflict of interest.
